# Impact of Atypical Hyperplasia at Surgical Margins on breast cancer outcomes in patients treated with neoadjuvant chemotherapy

**DOI:** 10.3389/fonc.2023.1202689

**Published:** 2023-05-19

**Authors:** An Su, Jing Zhang, Jieqiong Liu, Yaping Yang, Zhou He, Haoshi Bao, Heran Deng, Jiannan Wu

**Affiliations:** ^1^Guangdong Provincial Key Laboratory of Malignant Tumor Epigenetics and Gene Regulation, Sun Yat-Sen Memorial Hospital, Sun Yat-Sen University, Guangzhou, China; ^2^Breast Tumor Center, Sun Yat-Sen Memorial Hospital, Sun Yat-Sen University, Guangzhou, China; ^3^Anesthesiology Department, Sun Yat-Sen Memorial Hospital, Sun Yat-Sen University, Guangzhou, China

**Keywords:** atypical hyperplasia, surgical margins, ipsilateral breast tumor recurrence, neoadjuvant chemotherapy, breast-conserving surgery

## Abstract

**Background:**

Women with atypical hyperplasia (AH) is associated with a higher risk of future breast cancer. However, whether AH found at margins in patients with breast-conserving surgery (BCS) after neoadjuvant chemotherapy (NAC) needs re-excision is not well-defined. The aim of the present study was to evaluate the impact of AH at the surgical margins on the local recurrence and survival outcomes in breast cancer patients treated with NAC and BCS.

**Methods:**

A retrospective analysis comparing patients who treated with NAC and BCS with AH at the margins to those without AH was performed.

**Results:**

598 patients were included in this study. The 5-year rates of ipsilateral breast tumor recurrence (IBTR) were 4.6% and 6.2% in patients with and without AH, respectively. No significant differences were observed among the two groups in terms of IBTR, DMFS, or OS. HER2 overexpressing breast cancer patients with severe AH at margins have a significantly higher risk of IBTR compared to those without severe AH.

**Conclusion:**

Our study suggests that the presence of AH at the surgical margins of BCS in patients who received NAC does not appear to increase the risk of ipsilateral breast cancer. Therefore, there is no need for surgeons to routinely perform additional re-excision of AH found at the margins of BCS in these patients. However, selective re-excision should be considered in certain cases, particularly in patients with HER2 overexpression.

## Introduction

Neoadjuvant chemotherapy (NAC) is frequently used in early-stage breast cancer patients to reduce tumor size and convert them to candidates for breast-conserving surgery (BCS) (1, 2). BCS requires obtaining a negative margin, as positive margins (defined as ink on ductal carcinoma *in situ* or invasive carcinoma) increase the risk of local recurrence by two-fold (3).. Several factors are associated with an increased risk of local recurrence after BCS, including lymphovascular invasion, large tumor size, positive nodal status, extensive intraductal component, close or involved margin status, and negative hormone receptor status (4). Atypical hyperplasia (AH) of the breast, which includes atypical ductal hyperplasia (ADH) and atypical lobular hyperplasia (ALH), is a premalignant lesion that is not abnormal enough to be classified as carcinoma in situ (5). Patients with AH on breast biopsy of benign lesions have an approximate four-fold increased risk of later breast cancer (6, 7).

It is unclear whether AH at margins can lead to increased local recurrence in patients who underwent BCS, particularly in those who received NAC. Some studies have explored the issue but have produced conflicting results (8–10). Lennington et al. found that ADH is often located at the periphery of ductal carcinoma *in situ* (DCIS), which means that AH identified at the margin of a BCS specimen may represent a DCIS component already very close to the tumor margin (11). This close margin may be related to higher local recurrence of the breast (4). Therefore, it is necessary to investigate further whether AH at margins is associated with local recurrence of breast cancer. Our previous study reported on 244 breast cancer patients without NAC treated with BCS between 2009 and 2011, and we found that patients with AH at the margins experienced the same local control as those without AH (12). However, patients treated with NAC were excluded from our previous study. To address this gap, we conducted a study to evaluate the impact of AH at the surgical margins on local recurrence and survival outcomes in breast cancer patients treated with NAC and BCS. Therefore, this study aims to investigate the relationship between AH at surgical margins and local recurrence in breast cancer patients who received NAC and underwent BCS, the results will help to inform clinical practice and improve patient outcomes.

## Methods

### Patients

Institutional databases were reviewed to identify stage I-III breast cancer patients who underwen NAC and BCS at Sun Yat-sen Memorial Hospital, Sun Yat-sen University from 2009 to 2020. The study was approved by the Institutional Review Board (IRB) (SYSECKY-KS-2020-116) at Sun Yat-sen Memorial Hospital. All patients were diagnosed with invasive breast carcinoma through core needle biopsy. Clinicopathologic data including demographics, clinical oncologic features (tumor size, nodal stage) and tumor complete receptor information (ER, PgR, and HER2) was collected. The clinical and pathologic stages were defined according to the 8^th^ edition of the American Joint Committee on Cancer guidelines. Clinical stage was determined by ultrasound of the breast and lymph nodes. Lymph nodes with an abnormal appearance on ultrasound were routinely evaluated with a core-needle biopsy. A cutoff of 1% was used to determine the HR status on the core biopsy specimens. Tumors were defined as HER2 positive if they were 3+ by immunohistochemistry or demonstrated gene amplification by fluorescence *in situ* hybridization (13). Overall pathologic complete response (pCR) was defined as no residual invasive cancer in the breast or axillary lymph nodes.

### Treatment and pathologic considerations

All of the patients received a complete course of NAC consisting of taxane, anthracycline, or both, with trastuzumab was given to patients with HER2+ breast cancer. After completing neoadjuvant chemotherapy, patients underwent BCS, axillary staging surgery with sentinel lymph node biopsy (SLNB) and/or axillary lymph node dissection (ALND), and irradiation therapy. During lumpectomy, we removed the tumor with an approximate 1 cm grossly negative margin. To ensure negative margins, the cavity walls were shaved, and an intraoperative frozen section analysis was performed, which was described previously by Chen K et al (14). Any involved margins were excised until free margins were obtained, with the tumor-free margin defined as a negative margin, AH but no tumor at the margin was also defined as a negative margin. Postoperative paraffin-embedded hematoxylin and eosin (H&E) staining confirmed pathology diagnosis of the margin specimens. Two dedicated pathologists reviewed tumor-free margin specimens to verify negative margin diagnosis. Additional adjuvant chemotherapy, targeted therapy (trastuzumab), and endocrine therapy were given when necessary according to the NCCN guidelines. In this study, we analyzed atypical ductal hyperplasia (ADH) and atypical lobular hyperplasia (ALH) together as “atypical hyperplasia” (AH) due to their similar frequency and similar risk of breast cancer (7). Both ADH and ALH were diagnosed based on established criteria by Page et al (15). ADH involves distended ducts filled with monotonous epithelial cells forming complex patterns, while ALH features expanded lobular acini filled with small, round or polygonal cells lacking cohesion and acinar lumens (7).

### Statistical analysis

The primary end point of the present study was ipsilateral breast tumor recurrence (IBTR), defined as recurrence in the ipsilateral breast. The time to IBTR was calculated from the date of BCS to the occurrence of IBTR, and it was censored at the time of the last follow-up or the time of death among patients who did not suffer IBTR. Patients characteristics of the two groups were compared using the chi-square test and the Kruskal–Wallis test where appropriate. Kaplan–Meier method was used to estimate IBTR-survival, distant-metastasis-free survival (DMFS), and overall survival (OS). The log-rank test was used to assess differences between the groups. Multivariate analyses were performed using the Cox proportional hazards model. All values were two sided, and statistical significance was defined as *P*<0.05. All calculations were conducted using SPSS 19.0 software (SPSS Inc., IBM, Chicago, USA).

## Results

A total of 598 patients were included in this retrospective study, among whom 301 patients had AH at the margins in BCS, while 297 patients did not have this pathological feature at the margins. The median follow-up was 46 months (range 13-159). Clinicopathological characteristics and neoadjuvant chemotherapy response rate were compared between the two groups, and the results are presented in **Table 1**. There were no significant differences between the two groups in terms of age, tumor histologic type, initial clinic T stage, initial nodal stage, receptor status, post-neoadjuvant chemotherapy (NAC) pathologic T stage, post-NAC pathologic nodal status, or overall pathological complete response rate. For the 503 patients who had residual tumor after neoadjuvant chemotherapy, including invasive and *in situ* cancers, 77 cases required re-resection due to the presence of invasive or *in situ* cancer components in the margin tissue, the re-excision rate was 15.3%.

**Table 1:** Clinicopathlogical characteristics of two groups.

	Atypical hyperplasia	No atypical hyperplasia	*p-*value
Median age	43 years (rang 24-68)	43 years (rang 19-71 )	
Age (yr)	301	297	0.583
≤40	105 (34.9%)	110(37.0%)	
>40	196 (65.1%)	187 (63.0%)	
Tumor histology			0.770
Invasive ductal carcinoma	281 (93.4%)	279 (93.9%)	
Other type of invasive carcinoma	20(6.6%)	18(6.1%)	
Initial clinic T stage			0.554
cT1	24(8.0%)	31 (10.4%)	
cT2	221 (73.4%)	215 (72.4%)	
cT3-4	56 (18.6%)	51 (17.2%)	
Initial nodal stage			0.825
cN0	121 (40.2%)	119 (40.0%)	
cN1	167 (55.5%)	168 (56.6%)	
cN2-3	13 (4.3%)	10 (3.4%)	
Receptor status			0.739
HR+/HER2-	161 (53.4%)	148 (49.8%)	
HR+/HER2+	55(18.2%)	54 (18.1%)	
HR-/HER2+	42 (13.9%)	44(14.8%)	
HR-/HER2-	43 (14.2%)	51 (17.1%)	
ypT stage			0.622
ypT0	64 (21.2%)	73 (24.6%)	
ypT1	169 (56.2%)	161 (54.2%)	
ypT2	68 (22.6%)	63 (21.2%)	
ypNodal status			0.968
ypN0	162 (53.8%)	158(53.2%)	
ypN1	100(33.2%)	95(32.0%)	
ypN2-3	39(13.0%)	44(14.8%)	
Overall pCR			0.938
yes	55(18.2%)	55(18.5%)	
no	246(81.7%)	242(81.4%)	

Bold value is statistically significant when p < 0.05.

HR, hormone receptor; HER2, human epidermal growth factor receptor 2; pCR, pathologic complete response; NAC, Neoadjuvant chemotherapy.

**Figure 1:**
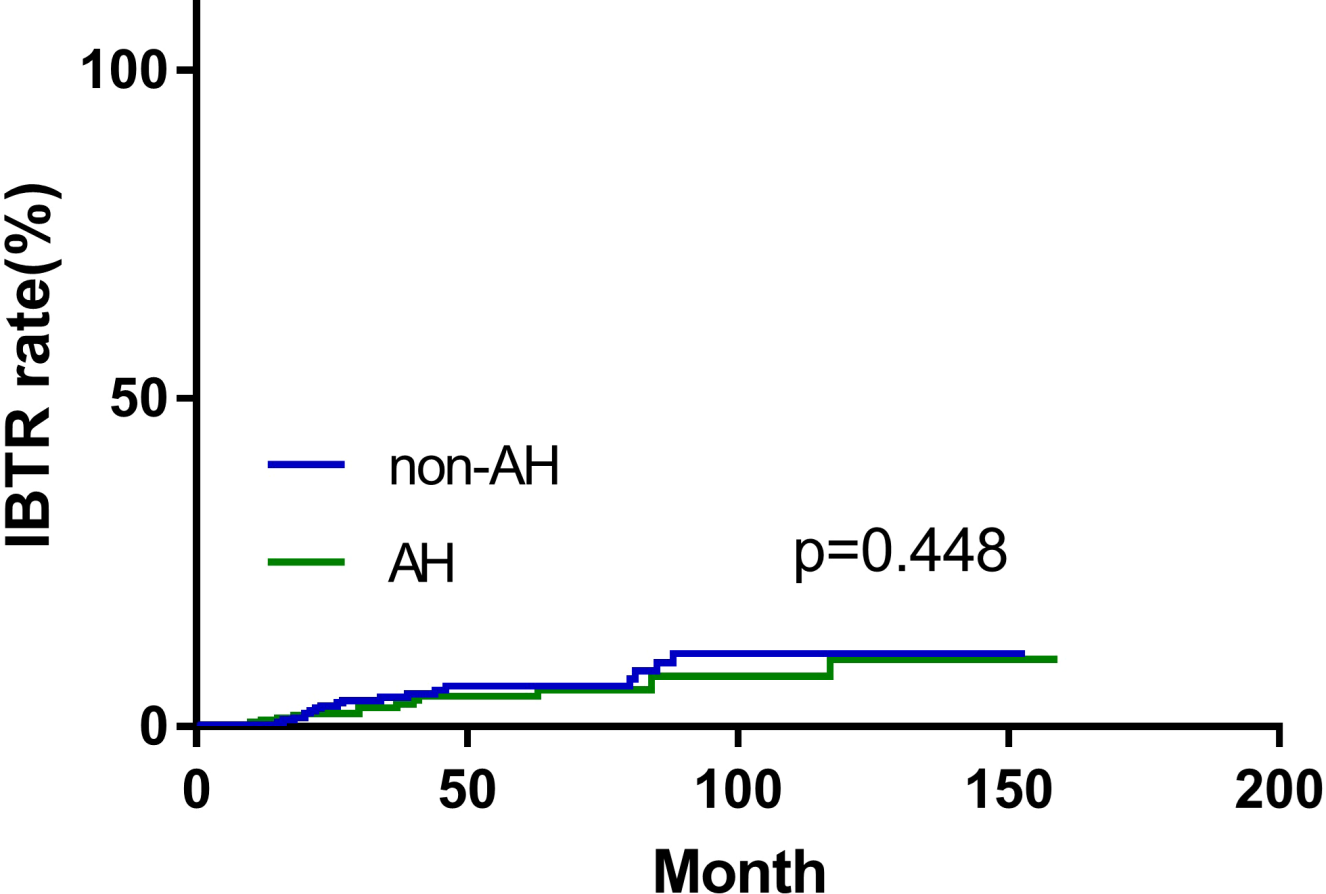
Ipsilateral breast tumor recurrence (IBTR) of patients with and without atypical hyperplasia.

**Figure 2:**
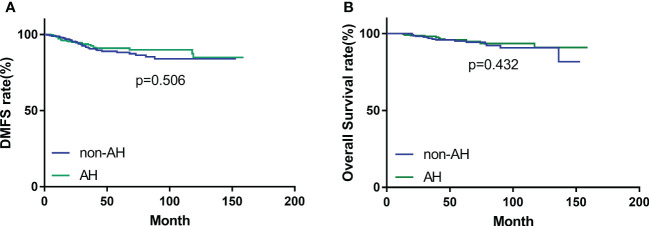
**(A)** Distant-metastasis-free survival (DMFS) and **(B)** overall survival (OS) of patients with and without atypical hyperplasia.

During the follow-up period, 14 (4.7%) patients in the AH group, and 19 (6.4%) patients in the non-AH group experienced ipsilateral breast tumor recurrence (IBTR). The 5-year rates of IBTR were 4.6% (95% CI, 3.2%~6.0%) and 6.2% (95% CI, 4.6%~7.8%) in patients with and without AH, respectively. The 5-year distant-metastasis-free survival (DMFS) rate was 91.0% (95% CI, 89.1%~92.9%) in the AH group and 88.0% (95% CI, 85.8%~90.2%) in the non-AH group, respectively. Additionally, the 5-year overall survival (OS) rate for patients with or without AH was 95.9% (95% CI, 94.5%~97.3%) and 95.2% (95% CI, 93.7%~96.7%), respectively. No significant differences were observed between the two groups of patients in terms of IBTR (**Figure 1**), DMFS (**Figure 2A**), or OS (**Figure 2B**) (*p* = 0.448, 0.506 and 0.432, respectively).

AH can be further classified into mild, moderate, and severe categories which border on ducal carcinoma in situ. Coopey et al. evaluated breast cancer events in a retrospective cohort of 2938 women with ADH, ALH, LCIS, and severe ADH, and estimated a 10-year risk of breast cancer of 17% for women with AH and 26% for women with severe ADH (16). Therefore, severe AH may be regarded as “higher level of risk” lesion. We further analyzed the outcomes between patients with severe AH (n=90) and those without AH (n=508) and found no significant differences in IBTR, DMFS, or OS (*p* = 0.138, 0.340 and 0.892, respectively). Among the 195 cases of HER2 overexpressing breast cancer, which included both HR+/HER2+ and HR-/HER2+ subtypes, 174 cases did not exhibit severe AH. Of these 174 cases, 11 patients experienced ipsilateral breast tumor recurrence (IBTR). However, in the remaining 21 patients who exhibited severe AH, 4 patients developed IBTR. These findings suggest that the risk of IBTR is significantly higher in patients with HER2 overexpression who exhibit severe AH at the margins compared to those without AH (p=0.039). However, this phenomenon was not observed in HR+/HER2- or triple-negative breast cancer (p=0.660 and 0.434, respectively).

It has been reported previously that some clinical, pathologic, and molecular factors were associated with IBTR after BCS (4). Therefore, we conducted an univariate analysis to identify clinical, pathological, and molecular factors associated with IBTR, DMFS, and OS. There was no significant association between AH status and IBTR in the univariate analysis (**Table 2**). Similarly, other clinical and pathological features, including age, tumor histologic type, initial clinic T stage, initial nodal stage, receptor status, post-NAC pathological T stage, post-NAC pathological nodal status, or overall pCR rate, were not significantly associated with IBTR (**Table 2**). On univariate analysis, patients with negative lymph nodes had better DMFS, and OS (**Table 2**).

**Table 2:** Univariate analysis of factors predicting IBTR/DMFS/OS in patients undergoing BCS.

Characteristics	Ipsilateral breast tumor recurrence		Distant-metastasis-free survival		Overall survival	
	Univariate		Univariate		Univariate	
	OR (95% CI)	*p*-value	OR (95% CI)	*p*-value	OR (95% CI)	*p*-value
Age (yr)		0.18		0.19		0.29
≤40	1		1		1	
>40	0.63(0.32-1.25)		0.70(0.42-1.165)		1.55(0.68-3.53)	
Tumor histology		0.56		0.95		0.99
Invasive ductal carcinoma	1		1		1	
Other type of invasive carcinoma	0.705(0.215-2.31)		0.97(0.35-2.67)		0.99(0.23-4.19)	
Initial clinic T stage		0.68		0.31		0.98
cT1	1		1		1	
cT2	0.82(0.30-2.59)		1.23(0.48-3.14)		0.90(0.15-2.67)	
cT3-4	1.30(0.37-4.54)		1.90(0.68-5.31)		0.88(0.22-3.61)	
Initial nodal stage		0.12		0.178		0.24
cN0	1		1		1	
cN1	2.10(0.94-4.70)		1.29(0.77-2.23)		1.58(0.69-3.64)	
cN2-3	3.68(0.78-17.43)		2.73(0.93-7.80)		3.57(0.75-16.92)	
Receptor status		0.055		0.64		0.347
HR+/HER2-	1		1		1	
HR+/HER2+	1.56(0.59-4.07)		0.70(0.33-1.50)		0.16(0.022-1.22)	
HR-/HER2+	3.05(1.31-7.10)		0.70(0.30-1.67)		0.000(0.000)	
HR-/HER2-	0.92(0.30-2.79)		0.70(0.33-1.52)		0.75(0.28-1.97)	
ypT stage		0.78		0.774		0.44
ypT0	1		1		1	
ypT1	0.75(0.34-1.69)		1.23(0.62-2.42)		1.91(0.65-5.61)	
ypT2	0.79(0.30-1.13)		1.32(0.60-2.92)		1.31(0.35-4.89)	
ypNodal status		0.7		0.029		0.013
ypN0	1		1		1	
ypN1	0.89(0.41-1.94)		1.97(1.11-3.49)		2.86(1.14-7.18)	
ypN2-3	1.37(0.54-3.48)		2.237(1.10-4.55)		4.40(1.60-12.14)	
pCR		0.75		0.27		0.18
no	1		1		1	
yes	0.86(0.33-2.23)		0.64(0.29-1.41)		0.37(0.090-1.58)	
AH status		0.5		0.4		0.51
non-AH	1		1		1	
AH	0.79(0.40-1.58)		0.80(0.48-1.34)		0.78(0.37-1.65)	

OR, odds ratio; CI, confidence interval; pCR, pathologic complete response; AH, atypical hyperplasia; BCS, breast-conserving surgery; IBTR, ipsilateral breast tumor recurrence; DMFS, distant-metastasis-free survival; OS, overall survival.

## Discussion

In this study, we aimed to expand on our previous conclusions by examining the outcomes of patients who received NAC and BCS. Our findings suggest that AH at the margins does not lead to worse outcomes in terms of local ipsilateral breast recurrence-free survival, disease-free survival (DMFS), or overall survival (OS) in patients who underwent BCS after NAC. The observed rate of ipsilateral breast tumor recurrence (IBTR) was low (33/598, 5.5%).

Previous research from the Netherlands has shown higher rates of tumor-involved margins in patients treated with NAC and BCS compared to those treated with primary BCS (23% vs. 10%) (2). A prospective study led by Elisabeth and colleagues found that removing additional tissue from the cavity shave margins during breast conserving therapy can significantly reduce the rates of positive margins and re-excision in patients (17). Our study identified a lower re-excision rate (15.3%, 77/503) compared to that reported in the Netherlands research, which could potentially be attributed to the utilization of the cavity shave method for margin assessment in our study.

Moreover, previous research by Coopey et al. found that patients with AH and severe AH were equally likely to develop invasive cancer and ductal carcinoma *in situ* (DCIS) (16). Nizre et al. (18) conducted a significant survey to determine the current management of atypical ductal hyperplasia (ADH) among American Society of Breast Surgeons (ASBrS) members. The researchers revealed that 61% of the surgeons favored no further surgery, while 30% recommended selective re-excision. Interestingly, the level of training had an impact on the response tendencies towards no further surgery. For instance, among cancer center surgeons, 80% would recommend no further surgery, 20% would suggest selective re-excision, and none advocated routine re-excision when ADH involved the margin. However, there are no established criteria for determining which patients would benefit from a re-excision procedure. In our study, we found that HER2 overexpressing breast cancer patients with severe AH at the margins had a higher rate of ipsilateral breast recurrence compared to those without severe AH at the margins. We believe that the following factors may contribute to these results. The use of trastuzumab in HER2-positive breast cancer often results in a significant reduction in tumor size. However, 71% of cases exhibit a scattered pattern of regression (19). Additionally, the presence of severe atypical hyperplasia at the margin of HER2-positive breast cancer in the surrounding tissue may suggest the possible existence of discrete tumor cells located just beyond the edge. Failing to perform a sufficiently wide resection may result in residual tumor. Our findings suggest that HER2 overexpressing breast cancer patients with severe AH at the margins may benefit from re-excision of the affected margins.

While AH is known to contribute to a higher risk of breast cancer, the present study did not find it to confer a higher risk of ipsilateral breast tumor recurrence (IBTR). This could be due to several reasons. First, the conclusion that AH contributes to a higher risk of breast cancer was drawn from people with benign diseases and not from patients who have already suffered from breast cancer. Patients with breast cancer have significantly different tumor burden than those with only benign disease. For example, in a study by Holland et al., of the 282 patients with invasive cancers who underwent lumpectomy and got negative margins, 105 (37%) showed no other tumor foci in the residual breast, but other tumor foci were found in the remaining 177 breasts, of which 20% of tumor foci were present within 2 cm of the reference tumor in the residual breast (20). Therefore, a negative margin does not indicate the absence of residual cancer in the breast, but the residual tumor can be controlled by radiotherapy and adjuvant treatments such as endocrine therapy and targeted therapy. The risk of IBTR after breast-conserving therapy is about 0.5–2% per year (21, 22), with an increased risk during the first few years, and the median time to IBTR is 36 months (23). Additionally, results from NSABP B-18 suggested that patients downstaged after NAC for BCS may have a higher local recurrence rate (24). Recent reports with long-term follow-up have demonstrated that the absolute risk of developing breast cancer is in the range of 1-2% per year (7, 25). More recently, Menes and colleagues’ research, which was large and contemporary, found that the 10-year cumulative risk of developing breast cancer in women with atypical ductal hyperplasia (ADH) is only 5.6% (26). Therefore, the risk of breast cancer due to AH is not higher than the risk of local recurrence associated with the possible residual tumor burden after BCS. Thus, it is not surprising that AH involved at the margin does not contribute to a higher risk of IBTR. Second, endocrine therapy can reduce IBTR. Data from the NSABP P-1 trial showed that tamoxifen administered for 5 years decreased the risk of invasive and non-invasive breast cancer by approximately 50% (27). In the present study, over 70% of the patients received endocrine therapy. We postulate that this result is partly attributable to the use of endocrine therapy. Third, the follow-up period of our study may not have been long enough to reveal the effectiveness of the difference between groups. Page et al. reported that the interval between ADH and ALH to breast cancer is 8.2 and 11.9 years (28), respectively, but the median follow-up period of our study was approximately 48 months, although the peak period for local recurrence is within three years (23).

However, our study has some limitations due to its retrospective design. The chemotherapy regimens used in our study varied among different subtypes of breast cancer, which may have influenced the relationship between NAC response and DFS/OS. Additionally, the number of patients enrolled in our study was limited. Future multi-centered studies with larger sample sizes and longer follow-up periods are needed to increase statistical power and provide a more definitive conclusion.

In summary, our study found no evidence of an increased risk of ipsilateral breast tumor recurrence (IBTR), distant metastasis, or mortality in patients with AH involving surgical margins who underwent BCS after NAC. Additionally, univariate analysis did not reveal any association between AH status and IBTR, distant metastasis-free survival (DMFS), or overall survival (OS). While further research is necessary to fully elucidate the relationship between AH status and IBTR in this patient population, our findings suggest that involvement of AH at the margins following NAC may be acceptable for appropriately selected patients with breast cancer. However, patients with HER2 overexpressing breast cancer and severe AH at the margins have a higher risk of ipsilateral breast cancer recurrence. Therefore, these patients may require further re-excision procedures to remove the affected margins and minimize the risk of recurrence.

## Data availability statement

The raw data supporting the conclusions of this article will be made available by the authors, without undue reservation.

## Ethics statement

The studies involving human participants were reviewed and approved by the Institutional Review Board (IRB) (SYSECKY-KS-2020-116) at Sun Yat-sen Memorial Hospital. Written informed consent for participation was not required for this study in accordance with the national legislation and the institutional requirements.

## Author contributions

Contributions: (I) Conception and design: JW, HD. (II) Administrative support: JL. (III) Provision of study materials or patients: AS, JZ. (IV) Collection and assembly of data: JZ, ZH, HB. (V) Data analysis and interpretation: YY, JL. (VI) Manuscript writing: JW, HD. (VII) All authors reviewed the manuscript. All authors contributed to the article and approved the submitted version.
